# The challenges of the expanded availability of genomic information: an agenda-setting paper

**DOI:** 10.1007/s12687-017-0331-7

**Published:** 2017-09-26

**Authors:** Pascal Borry, Heidi Beate Bentzen, Isabelle Budin-Ljøsne, Martina C. Cornel, Heidi Carmen Howard, Oliver Feeney, Leigh Jackson, Deborah Mascalzoni, Álvaro Mendes, Borut Peterlin, Brigida Riso, Mahsa Shabani, Heather Skirton, Sigrid Sterckx, Danya Vears, Matthias Wjst, Heike Felzmann

**Affiliations:** 1 Centre for Biomedical Ethics and Law, Department of Public Health and Primary Care, KU Leuven, Leuven, Belgium; 2 Leuven Institute for Human Genomics and Society, 3000 Leuven, Belgium; 3 Faculty of Medicine, University of Leuven, Leuven, Belgium; 4 Centre for Medical Ethics, Faculty of Medicine, University of Oslo, Oslo, Norway; 5 Norwegian Research Center for Computers and Law, Faculty of Law, University of Oslo, Oslo, Norway; 6 Norwegian Cancer Genomics Consortium, Oslo, Norway; 7 Centre for Medical Ethics, Institute of Health and Society, University of Oslo, P.O Box 1130, Blindern, 0318 Oslo, Norway; 8 Cohort Studies, Norwegian Institute of Public Health, Oslo, Norway; 9 Department of Clinical Genetics, Section of Community Genetics, Amsterdam Public Health Research Institute, VU University Medical Center, Amsterdam, the Netherlands; 10 Centre for Research Ethics and Bioethics, Uppsala University, Uppsala, Sweden; 11 Centre of Bioethical Research and Analysis (COBRA), National University of Ireland (Galway), Galway, Republic of Ireland; 12 RILD Building, Royal Devon and Exeter Hospital, University of Exeter Medical School, Exeter, UK; 13 EURAC Research, Bolzano, Italy; 14 i3S, Instituto de Investigação e Inovação em Saúde, IBMC-UnIGENe and Centre for Predictive and Preventive Genetics, Universidade do Porto, Porto, Portugal; 15 Clinical Institute of Medical Genetics, University Medical Center Ljubljana, Šlajmerjeva 4, 1000 Ljubljana, Slovenia; 16 Instituto Universitário de Lisboa (ISCTE-IUL), CIES-IUL, Lisbon, Portugal; 17 Faculty of Health and Human Sciences, University of Plymouth, Drake Circus, Plymouth, PL4 8AA UK; 18 Bioethics Institute Ghent, Ghent University, Blandijnberg 2, 9000 Ghent, Belgium; 19 Helmholtz Center Munich, National Research Centre for Environmental Health, Institute of Lung Biology and Disease, Munich, Germany; 20 Institute of Medical Statistics, Epidemiology and Medical Informatics, Technical University Munich, Munich, Germany

**Keywords:** Genomics, Clinical and research genomic data, Return of results, Data sharing, Informed consent, Direct-to-consumer genetic testing

## Abstract

Rapid advances in microarray and sequencing technologies are making genotyping and genome sequencing more affordable and readily available. There is an expectation that genomic sequencing technologies improve personalized diagnosis and personalized drug therapy. Concurrently, provision of direct-to-consumer genetic testing by commercial providers has enabled individuals’ direct access to their genomic data. The expanded availability of genomic data is perceived as influencing the relationship between the various parties involved including healthcare professionals, researchers, patients, individuals, families, industry, and government. This results in a need to revisit their roles and responsibilities. In a 1-day agenda-setting meeting organized by the COST Action IS1303 “Citizen’s Health through public-private Initiatives: Public health, Market and Ethical perspectives,” participants discussed the main challenges associated with the expanded availability of genomic information, with a specific focus on public-private partnerships, and provided an outline from which to discuss in detail the identified challenges. This paper summarizes the points raised at this meeting in five main parts and highlights the key cross-cutting themes. In light of the increasing availability of genomic information, it is expected that this paper will provide timely direction for future research and policy making in this area.

## Introduction

Rapid advances in microarray and sequencing technologies are making genotyping and genome sequencing more affordable and readily available. The decreasing cost and time needed for sequencing has generated the expectation that the use of next-generation sequencing technologies (NGS) (i.e., new high-throughput and massively parallel DNA-sequencing technologies) will greatly increase in a wide range of contexts (Rehm 2017). Already, NGS is increasingly used to identify causative mutations in some patients with rare or undiagnosed diseases of genetic origin (Levenson 2014). Furthermore, the expectation has grown that genomic-sequencing technologies could be applied in a broad range of clinical situations, leading to personalized diagnoses and personalized drug therapy. Data arising from genome sequencing is likely to lead to a better prediction of disease risk and treatment response and the avoidance of adverse events (Lazaridis et al. 2016; Rehm 2017; Soden et al. 2014; van Zelst-Stams et al. 2014).

Furthermore, it is anticipated that an increasing number of healthy individuals will use genomic technologies to predict personal risks (Knoppers et al. 2014; van El et al. 2013). For over a decade now, genetic testing companies have been marketing and selling genetic tests direct to consumer (DTC) via the internet (Howard and Borry 2012). A number of online interpretation services (such as Promethease, LiveWello, and Interpretome) have also emerged that allow consumers to receive an analysis of their own raw genomic data received from these DTC genetic testing companies (Badalato et al. 2017). These online services will allow for further interpretation of the user’s genome.

Between 2013 and 2017, the COST Action IS1303 “Citizen’s Health through public-private Initiatives: Public health, Market and Ethical perspectives” identified and reunited a community of academic and industry researchers as well as other stakeholders with expertise in bioethics, social studies of science and technology, genetics, information and communication technology, stakeholder deliberation, and patient-centered initiatives (PCI). As part of this networking project, a meeting was convened in Leuven (Belgium) on 21 and 22 March 2016, in order to identify and discuss the challenges related to the expanded availability of genomic information in society. A particular focus was placed on the context of public-private partnerships in genomics. The meeting aimed to promote a mutually informative and collaborative agenda-setting process. The aim of this document is to identify, via horizon scanning, the main forthcoming challenges and areas of interest arising from the availability of genomic information in society. It is expected that the results of this paper will allow for constructive reflection on future developments and the identification of research priorities. It is designed for use by a wide array of stakeholders, such as regulators, policy makers, healthcare institutions, patient organizations, and industry.

**Fig. 1:**
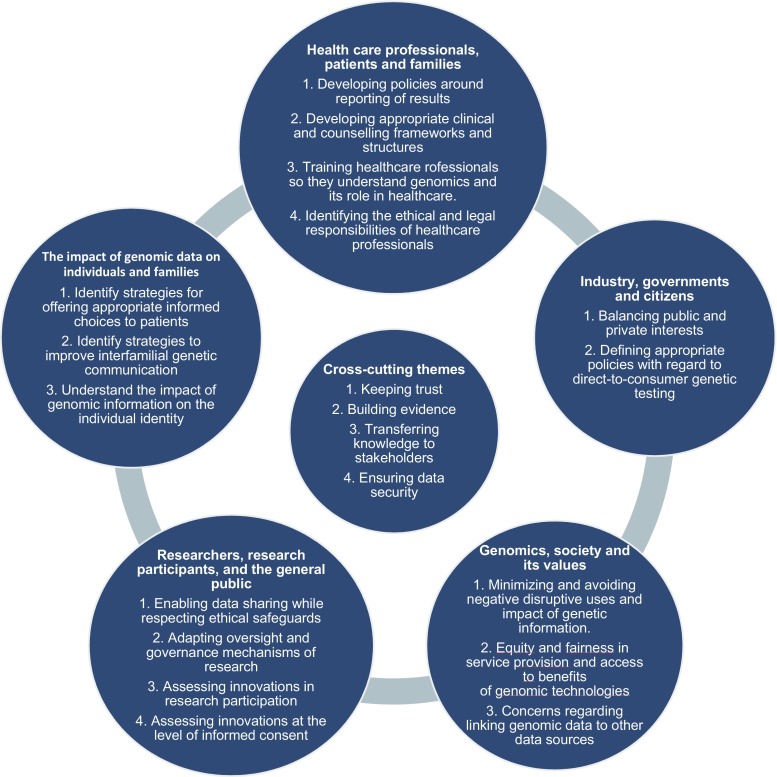
Five salient/key relationships in the realm of genetics and genomics and the central cross-cutting themes

Current and future challenges were identified in the context of five salient/key relationships in the realm of genetics and genomics (Fig. 1): (1) healthcare professionals, patients, and families; (2) genomic data and its impact on individuals and families; (3) researchers, research participants, and the general public; (4) genomics, society, and its values; and (5) industry, governments, and citizens. An overlap between these different relationships obviously exists, but they help to frame the various areas of focus. As well as these overlaps, some identified challenges are also relevant to more than one type of relationship.

## Healthcare professionals, patients, and families

### Developing policies for reporting results

The clinical implementation of NGS technologies creates huge challenges for laboratories and clinicians at the level of returning results. The use of NGS for whole exome or whole genome sequencing has the potential to identify variants in genes for which the function is unknown or variants for which the pathogenicity has not been established (Ream and Mikati 2014). Some commentators have concluded that using NGS may “raise more questions than it answers for some patients” (Ream and Mikati 2014). In addition to issues related to the interpretation and reporting of these variants of uncertain significance (VUS), uncertainty remains regarding how to deal with incidental findings unrelated to the clinical indication of the test. This issue is particularly complicated when the variants relate to late-onset conditions (Katsanis and Katsanis 2013) or untreatable conditions (Vasta et al. 2012). Such information can also have familial implications (Babkina and Graham 2014). Different guidelines and protocols that describe how to handle the return of results, including VUS and incidental findings, have been developed and need further elaboration as well as potential harmonization, especially with regard to the pertinent responsibilities of involved parties (Vears et al. 2017a, b).

### Developing appropriate clinical and counseling frameworks and structures

The enhanced technical options for genetic testing are not yet accompanied by comprehensive genetic counseling models for the genomic era. New models and frameworks of genetic counseling that extend beyond the traditional clinical genetics and genetic counseling setting need to be developed (Bradbury et al. 2014). Given the potential of NGS to generate high volumes of data, and uncertainties around results of the data generated, there is a pressing need to revitalize current genetic counseling services. Furthermore, individuals receiving sequencing results may adopt different roles such as patient, customer, hobbyist, or activist. Previously, individuals largely had a unique and defined pathway for accessing genetic information through the traditional healthcare setting (via clinical geneticists and/or genetic counselors) on the basis of specific clinical concerns or family history. In contrast, individuals now have the opportunity to choose genetic testing without the intermediary of a professional assessment of clinical need and can obtain testing for a variety of purposes, including mere curiosity. Individuals may also choose to use sequencing services that provide access to raw data without interpretation, providing them with “unfiltered” genetic information to use as they see fit. They could, for example, attempt to “self-interpret” with the support of publicly available sites for the analysis of genetic data (such as openSNP), or use it for entirely unrelated purposes, such as artistic endeavors (Werner-Felmayer 2014). Genetic counseling policies should be developed in relation to the different ways individuals can access genomic information. As part of this, it is important to (re)define the roles of clinical geneticists, genetic/genomic counselors, and other professionals, such as general practitioners specialized in clinical genetics who provide advice in relation to the wide array of genomic information (Middleton et al. 2015).

### Training healthcare professionals so they understand genomics and its role in healthcare

In the clinical setting, even among genetic experts, there is a clear need for a collaborative, multidisciplinary effort (biology, bioinformatics, clinical genetics) to interpret and understand NGS results. As genomics continues to move from specialized centers to mainstream medicine, various medical specialists who are unfamiliar with clinical genetics or genetic counseling may be increasingly required to have a greater role in the prescribing and/or interpretation of genetic testing and the communication of genomic information. For instance, Gen-Equip (Paneque et al. 2017; Primary Care Genetics) is an example of an effort that has been made to enable health professionals who are working in primary care to update their knowledge and skills in genetics. The Gen-Equip project (https://www.primarycaregenetics.org) was co-funded by the EU Erasmus+ Programme. It developed a program of online learning modules and tools to support daily practice in primary care about genetics.

It will be necessary to educate and train healthcare professionals to translate this changing landscape into appropriate patient care, including being family-centered. Authors have identified a need for a new kind of physician who will be trained in several disciplines including medicine, genetics, and counseling (Gonzalez-Garay et al. 2013; Iacobazzi et al. 2014). Others advocate either for clinical geneticists to have a more prominent role in the clinical interpretation of data (Gomez-Lobo 2014; Grody et al. 2013) or for several experts such as “molecular biologists, clinical geneticists, and bioinformaticists” to combine their efforts for data interpretation (Grody et al. 2013). The implementation of NGS is no longer viewed as an individual physician’s endeavor, and therefore clinics offering genomic testing will need to adapt to this increased need for cross-disciplinary collaboration (Rigter et al. 2013), including conducting ethical, legal, and social issues research to accompany the clinical advances, especially while roles for laboratory geneticists and clinicians are changing.

### Identifying the ethical and legal responsibilities of healthcare professionals towards families

Healthcare professionals are increasingly asked for advice about the communication of genetic risk information to individuals as well as regarding communication within families. Based on the premise of medical confidentiality, professional guidelines recommend that professionals should not contact a client’s family members directly (Forrest et al. 2007) without his or her approval. Adherence to this guideline means that the client’s wish to disclose (or not disclose) information to relatives, must be respected (Hodgson and Gaff 2013). However, these guidelines also state that professionals should actively encourage clients to transmit relevant risk information to relatives and support them throughout the communication process (Forrest et al. 2007). When clients fail to disclose important information to relatives, professionals are confronted with potential ethical tensions between, on the one hand, addressing the needs of the individual and his/her right to confidentiality, and on the other hand, considering the potential for harm to uninformed relatives (Dheensa et al. 2015a). Some have recommended a more proactive role for health professionals (Battistuzzi et al. 2012; Otlowski 2013), although there is lack of clarity regarding how this could be achieved. Legislative frameworks in countries such as France, Australia, and Norway have created mechanisms that provide healthcare professionals with the potential to override their patients’ confidentiality in the interests of their relatives (Dheensa et al. 2015b; D’Audiffret van Haecke and de Montgolfier 2016; Weaver 2016). It is important to study the impact of these legislative changes and to consider whether they should be implemented more widely. The fact that such large volumes of data can be generated about patients also raises the question of whether there is a duty for health professionals to re-contact former patients should new genomic findings of potential clinical relevance come to light (Carrieri et al. 2017b). Although disclosing these findings may offer novel and more effective diagnostic/clinical options to the patient, re-contact also has the potential to cause anxiety and alarm to recipients of this new information, and their families, and may be logistically very difficult to achieve in practice. This highlights the need to explore the attitudes of individuals regarding communication of risks to their families as well as the factors that influence them towards a course of action. This also raises questions about the level of confidence of health professionals in performing the proposed practices, the provision of necessary funding and resources for these activities, as well as the creation of the necessary infrastructure to accommodate said practices. This might include updated registries, patient portals, other forms of consent, mobilization of patients’ associations in order to sensitize patients to regularly contacting genetic services, providing ongoing training for the genetic counseling workforce, and being open to adopting novel approaches if needed (Carrieri et al. 2017a).

## The impact of genomic data on individuals and families

### Identifying strategies for offering appropriate, informed choices to patients

In light of the new potential applications arising from using NGS in healthcare, various challenges remain with regard to obtaining informed consent, the reporting of results, and the inclusion of patient preferences regarding the return of results (Budin-Ljøsne et al. 2016). Determining which results should be returned, including incidental findings and VUS, following the use of NGS for diagnostic purposes, poses challenges for laboratories and clinicians (see below). It also poses challenges for individuals and families in making (truly) informed decisions with regard to the results they wish to receive. Indeed, they may not have enough information and/or understanding to support such a truly informed decision. More research is required to develop appropriate strategies to explain the different types of results that could be generated, and the related uncertainties before a test. Research also needs to be performed regarding how best to report results to patients, including how to support probands to discuss, these results with family members (Daly et al. 2016; de Geus et al. 2016), if necessary. This approach should include discussion among different stakeholders, as well as careful consideration of the impact that reporting strategies could create in both patient populations and the general public, and with regard to the potential costs to the healthcare system. The access to genomic medicine will also increasingly be available throughout the lifespan, from conception to elderly care. Individuals will be confronted with increasing technological possibilities and related informed choices to be made in various types to situations, such as preconceptional carrier screening, prenatal testing, preimplantation genetic diagnosis, newborn screening, tumor profiling, or genomic risk assessments in adult life (Rehm 2017).

### Identifying strategies to support interfamilial genetic communication

Clinical genetic healthcare providers have always strongly emphasized the familial nature of genetic information, and this has, in turn, guided patients’ use of these genetic services. Emphasis has mainly been placed on helping the individual understand testing, obtaining consent, and returning the results of testing to the individual. Less attention has been given to how to help these individuals respond to their genetic information, particularly when considering the *shared* nature of genetic information. As genetic sequencing and testing also has implications for relatives, genetic healthcare services have the challenge of supporting families, not just individuals (Eisler et al. 2017). Sequencing whole genomes/exomes potentially increases the need to involve family members to clarify inconclusive test results (newly-discovered variants and variants of uncertain significance) (Hallowell et al. 2015). Therefore, more research is required to explore the following: how families cope with genetic information; to what extent barriers exist relating to the disclosure of genetic information within families; and how such information impacts interfamilial relations. Although patients might initially feel inclined to transmit genetic risk information to their relatives, in reality, sharing of this information can be problematic. Individual perspectives, patterns of family dynamics, disease characteristics, and cultural factors may cause individuals to withhold or delay the disclosure of genomic information to at-risk relatives (Daly et al. 2016; de Geus et al. 2016; Vos et al. 2011). It has been argued that genetic information pushes the boundaries of individual autonomy from pure independence to a more relational approach to family responsibility (Widdows 2013). Such approaches stress the balance between rights, responsibilities, and the autonomy of individuals dealing with their own genetic information and the way these considerations intertwine with those of a family (Dheensa et al. 2016). Patients may also be unsure of the responsibilities of the healthcare professionals who have been involved in their diagnosis—some patients believe that their clinician is responsible for informing their relatives, rather than the patient themself (Mesters et al. 2005).

### Understanding the impact of genomic information on individual identity

The increasing availability of genomic information, within and outside the context of the traditional healthcare system (i.e., via direct-to-consumer genetic testing companies), provides new opportunities for individuals to engage with this information (O’Riordan 2016). Individuals are now able to have their own genetic data interpreted by all kinds of third-party interpretation services, outside of a clinical context. Healthcare professionals will increasingly being challenged by requests from individuals to help interpret genetic information that was obtained outside a traditional context. This might put pressure on healthcare systems, as a lot of this information might be of limited clinical validity and utility and, in most of the cases, genetic testing was not on medical indication (McGuire and Burke 2011).

Moreover, genomic information opens up new avenues for integrating genomic information into individuals’ conceptions of “self” (Novas and Rose 2000). A “balancing” of the perceptions of one’s “genetic side” as compared with one’s “aspects of oneself” also has relevance not only for personal identity, but for expectations, concerns, hopes, and decisions regarding genetic/genomic information, technologies, and services. Genetic information may be perceived as an exceptional window into our deep identity or may be seen as just one of many sources of information about the “self.” Further research is needed to understand the impact of genomic information on patients and families both within and outside the healthcare system.

## Researchers, research participants, and the general public

### Enabling data sharing while respecting ethical safeguards

In order to facilitate public health research, a diverse group of international and national funders of health research agreed to promote “greater access to and use of data” in equitable, ethical, and efficient ways (Walport and Brest 2011). More specifically in genetics and genomics, international and national policies and guidelines have established general frameworks to guide researchers in their data-sharing endeavors (Expert Advisory Group on Data Access 2015; Human Genome Organisation 1996; National Institutes of Health 2014; The Organisation for Economic Cooperation and Development 2007). Biomedical journals have also increasingly made data sharing a condition of publication (Barbui 2016; Barsh et al. 2015). In order to enable scientific advances, various publications have argued for the identification and removal of practical, legislative, professional, institutional, and attitudinal obstacles in order to achieve large-scale creation, access, and integration of data with sufficient sustainability (Burn 2016; Majumder et al. 2016; Wilbanks and Friend 2016). Regarding sharing practices to facilitate downstream uses of data, it is important to ensure that the rights of all parties involved (namely members of the general public, research participants, and their families, researchers, and funding bodies) are respected (Williams and Pigeot 2017). Data sharing, and genomic data-intensive research in general, may trigger concerns that differ considerably from concerns regarding research with human participants, which traditionally tend to be associated with physical risks. In particular, processing sensitive genomic data may raise informational risks for the data subjects, their family members or ethnic groups. Use of genomic data in a discriminatory manner by third parties, such as insurance companies or employers, is a prime example of the unintended consequences of processing genomic data. Consequently, employing a tailored approach to protect the rights of research participants is necessary (Shabani et al. 2014). Data-sharing policies should create mechanisms to reinforce the accountability of the researchers and data users, thereby ensuring that robust procedures are in place to govern data sharing and to respond to data misuses in an adequate manner (Lemke et al. 2010; Trinidad et al. 2010). Policies should endeavor to establish transparent, fair, and objective access and sharing procedures in order to ensure responsible data sharing (Shabani et al. 2015a), and to avoid unintended secondary uses of the data (O’Doherty et al. 2016). At the moment, data-sharing policies are mostly developed within the context of research projects by funders (e.g., NIH, Wellcome Trust) but are often not harmonized across projects and have a limited outreach (Budin-Ljøsne et al. 2014). For instance, they often do not provide guidance on how data produced within a project should be governed after project completion (Bobrow 2015). Furthermore, data sharing for clinical data is needed for optimal interpretation of variants (Hayden 2012).

Importantly, sharing individual-level genomic data also fuels concerns regarding the privacy of data subjects (Rothstein 2010). Privacy breaches resulting from re-identification of data could lead to harm for individuals and undermine public trust on the robustness of the data protection measures adopted by research institutions. Furthermore, while stand-alone anonymized genomic information is currently difficult to re-identify, such re-identification is not impossible. That being said, to date, the reported incidence of re-identification of genomic data has been limited, often requiring high levels of expertise (Gymrek et al. 2013; Homer et al. 2008; Shringarpure and Bustamante 2015). Nevertheless, the evolving potential of genomics and bioinformatics makes the risks of re-identification and/or privacy breaches moving targets, thereby requiring ongoing monitoring of the field and assessment of the sufficiency of the pertinent legal, ethical, and practical safeguards in place. The importance of adopting organizational and technical safeguards has been highlighted in the recent General Data Protection Regulation (GDPR). While GDPR suggests technical measures such as pseuonymization as an example of safeguards, it is crucial to further elaborate the additional organizational and technical measures to safeguard research participants and patients in the view of sensitive health and genomic data processing.

### Adapting oversight and governance mechanisms for genomic research

Current models of research governance were created at a time when research was often conducted at one site, by one team and involved a limited number of participants. These days, much of research is often multi-sited, international (e.g., research consortia) and organizationally complex (Kaye 2011; Kaye and Hawkins 2014). Effective and flexible research governance models that are harmonized across jurisdictions are required to meet the needs of current research approaches. Mechanisms are needed that enable greater transparency and allow for a greater involvement of research participants (Homer et al. 2008; Kaye et al. 2015a; Williams et al. 2015). Data access oversight bodies are examples of new governance tools that might be able to ensure appropriate monitoring of secondary research uses of data (Shabani et al. 2015b). Data access committees could maintain oversight of downstream data uses which are not yet known at the time of data and sample collection. It is expected that oversight bodies play a key part in reassuring research participants that their data is in safe hands and being used in ways that benefit science and society or are consistent with the consent they have given. In doing so, oversight bodies should adopt fair, objective, and transparent access arrangements.

### Assessing innovations in research participation

The role of research participants in genomic research and data sharing is evolving (Dove et al. 2012). It has been argued that both research participants and researchers would benefit from the active involvement of participants in various steps of the research process, from data collection to the management of data access (Erlich et al. 2014), and also obtaining their input when developing research policies (Pomey et al. 2015). Some have argued that by using the potential of various online platforms, individuals’ ongoing interactions with researchers, research institutions and other participants would be facilitated. DNA.LAND, Free The Data, and Patients Like Me exemplify initiatives that enable a broad scope of research participation by individuals, including sharing personal genomic and health-related data. The potential challenges to research ethics principles of adopting such approaches require further exploration (Shabani and Borry 2015). Individuals should have sufficient understanding of the research procedure and the associated risks and benefits to ensure informed decision making (Pereira et al. 2014). In particular, concerns exist with regard to the sharing of genomic data with biotech and pharmaceutical companies (Roberts et al. 2017). Questions also exist with regard to the transparency of such data sharing, the appropriateness of used informed consent and the potential lack of ethics approval (Niemiec and Howard 2016).

### Assessing innovations at the level of informed consent

Ideally, consent for healthcare procedures is a dynamic process, with an emphasis on disclosure of relevant information to the client, and then assessing the client’s understanding of the information and their ability to communicate their consent (Appelbaum 2007). In practice, consent for genetic testing often involves a punctual/one off process whereby experts provide information to participants, who then sign a paper-based consent form. However, this approach may be insufficient to inform research participants about the scope of research and the associated risks and benefits (Hayden 2012). The perceived shortcomings of this approach have led some to conclude that the current consent process, including the forms, are insufficient, and thus adopting alternative approaches appears inevitable (Hayden 2012). Alternative models, such as dynamic consent, have been suggested in order to introduce more flexibility to the consent process (Budin-Ljøsne et al. 2017; Kaye et al. 2015b). While these new consent models have potentially beneficial aspects in addition to obtaining and maintaining valid consent, such as increased participant engagement (Teare et al. 2015), they still need further research and analysis (Mascalzoni et al. 2008).

## Genomics, society, and its values

### Minimizing and avoiding negative disruptive uses and impact of genetic information

Little is known about how individuals or societies at large deal with genomic testing information or how such information impacts social relations (for example, when information is found about predispositions to stigmatizing diseases such as mental disorders (Gershon and Alliey-Rodriguez 2013) or cancer (Tercyak et al. 2013)). Stigmatization based on genomic information, whether it is based on genomic markers for ethnicity or disease, is a concern and steps should be taken to ensure that genomic information is not disruptive at either the familial or societal levels. Genomic information may be used to discriminate against individuals and their families (for example, in the work place or by insurers) on the basis of their genetic profile/genetic risk predisposition. Cases already exist of discrimination based on information produced through the genetic screening of newborns (Levenson 2016). Some groups, such as ethnic minorities (Joly et al. 2014) and future generations/offspring, may be particularly exposed to genetic discrimination. Indigenous peoples can also be exposed to genetic stigma and discrimination, and mechanisms to mitigate this need to be developed (Arias et al. 2016). Finally, human rights infringements can occur in countries which aim to collect the DNA from all of their citizens in order to develop forensic databases (as exemplified by the recent case of Kuwait) (O’Doherty et al. 2016; Thielking 2016).

### Equity and fairness in service provision and access to benefits of genomic technologies

Recent developments have resulted in an increase in the number of genetic tests available and a decrease in the price of genome sequencing. Therefore, the number of people who could access and potentially benefit from genetic testing is larger than ever (Rehm 2017). However, few studies describe to what extent the population for whom clinical benefit can be achieved is adequately served. There is a possibility that only those people who can personally afford the testing, or who are included in research projects, would undergo testing, such as a relatively high proportion of highly educated people in affluent countries. This raises serious ethical issues around the inequality of access to genomic healthcare. Authors who describe the reduced cost of sequencing, such as the $1000 genome, rarely mention the additional human resource costs involved in interpretation and downstream clinical care (Morrison et al. 2014). Given the financial constraints in healthcare systems, if not all services/technologies can be covered, criteria should be developed to determine which genetic services or genetic testing technologies should be funded from public budgets (Severin et al. 2015). Prioritization of genetic testing should be based on considerations of medical benefit, health need, empowering life-time decision making, and costs (Severin et al. 2015). However, the demands of fairness and equity (as with concerns over inequalities of access) may be more complicated and in need of more carefully nuanced responses than may initially appear. There is a general underlying concern that is related to the idea that should differential access to genetic technologies be allowed for those who are able to pay, it would give rise to new forms of unfairness and unjust inequalities—indeed, a key concern for many is how it would affect the central notion of equality of opportunity in society. Nevertheless, simply restricting differential access may be problematic from the point of view of overall utility (leveling down where not accessible to all) and requiring equal or universal access (or even reasonable approximations of either in the near term) may not be feasible when we are talking about a highly expensive (and to many extents limited) good that has to be weighed against other priorities in any public budget (e.g., with regard to education, general healthcare, water treatment, infrastructure, housing, etc.) (Farrelly 2007). Conversely, while Crozier and Hajzler (2010) note that many would view market forces as conflicting with the public good, they also highlight the role of such forces in promoting this good by widening access to the technologies in question. The market, they suggest, would advance the access by those less well-off to genetic technology through the market stimulus achieved by the wealthy gaining such access at an earlier point (Crozier and Hajzler 2010). An ideal egalitarian scenario that would not give proper scope to the potential role of the private sector and of private incentives (usually via the notion of “profit”) could be an overtly romanticized idea (Farrelly 2007). Given the feasibility constraints of most western societies, with limited budgets and a costly technology (while reducing in cost, it is still relatively costly, especially taking into account all steps involved), including a role for the private sector, via a regulatory framework that permits some innovation-friendly incentive-based inequalities in access, may be the best approximation of long-term fairness and equity.

### Linking genomic data to other data sources

A particular concern about data use in genomics refers to the continuously developing possibilities of interpreting and understanding genomic information. Given the exponential growth in data storage capacities and computational infrastructure, the integration of genomic data into the vast amounts of existing data will provide additional opportunities to capture the significance of genomics for improvement of health. Data brokers, such as Axicom, and data holders, such as Google and Facebook, collect personal information about consumers, and then combine and analyze said data to make inferences about them, including potentially sensitive inferences. This may infringe the privacy of individuals and expose them to significant risks (for instance, because data brokers often store data indefinitely) (Federal Trade Commission 2014). Therefore, adopting adequate legal safeguards for privacy of the individuals and addressing pertinent issues, such as intellectual property and access by the third parties, will be of paramount importance.

Similarly, data brokers are paying attention to the potential uses of genomic data. The current largest data holders would be able to connect an analysis of genomic data to an extraordinarily fine-grained and comprehensive set of behavioral and social information arising from their pervasive services. Drawing on such a vast repository of “life world”-related information may allow previously unprecedented opportunities for the analysis and contextualization of genomic information. This will create opportunities for new knowledge and insight, as well as significant potential for abuse. One particular concern in this context is the impact of the availability of such information on data privacy. As vast quantities and types of data, including face and fingerprint recognition, keyboard typing or other web surfing habits, consumer characteristics, and genome predictions, are available to a large number of commercial stakeholders, these stakeholders can cross link distant data sources (Wjst 2010). Genomic information is likely to become part of that integrated picture, especially if it is shared via the Internet and outside protected spaces. Accordingly, genetic privacy is becoming increasingly less likely in the long-term. A general issue that this raises concerns the consequences of a shift in power whereby those who are gathering, cross-linking and analyzing the digital footprints of individuals may have more knowledge about the individual than the individual herself (Lupton 2015). While the unprecedented availability of this amount of data may be a type of “holy grail” for data researchers, it poses many ethical challenges that extend beyond the practical/technical challenges of the development of hardware capable of dealing with the amount of data. In addition, the increasing use of algorithms in the health care setting raise questions about accountability of the users and potential risks for the data subjects (Mittelstadt and Floridi 2016).

## Industry, governments, and citizens

### Balancing public and private interests

The past decade has witnessed the rapid development of genomics research. Industry has played an important role in both the development of genomic research and the translation from research to clinical practice (Zerhouni et al. 2007). Policy makers have endorsed collaborations between public and private partners with the goal of stimulating innovation and the economy, creating jobs, and achieving a faster implementation of new technologies (Department of Health UK 2013). However, the interaction between public and private actors is also associated with ethical and social challenges. Finding balances between public and private interests has been a long lasting difficulty in human genetics (Contreras 2014). Symbolic of this was the competition between the public consortium of the International Human Genome Project and the private company Celera Genomics, to see which could sequence the human genome first. Discussions have also revolved around genetic disease patents, such as the *Association for Molecular Pathology* vs. *Myriad Genetics (2013)* and the *Greenberg v. Miami Children’s Hospital Research Institute* cases (Sterckx and Cockbain 2016). Furthermore, various debates have developed about the access of commercial companies to population-based biobanks, such as deCODE genetics in Iceland (Árnason and Andersen 2013). In December 2016, academic institutions met in court to decide on gene editing patents, potentially worth billions. Although these various cases highlight different problems, they all illustrate the challenge of finding a balance between, on the one hand, stimulating research and innovation, and, on the other hand, promoting ethical values such as trustworthiness, respect for autonomy, transparency, and respect for confidentiality and privacy. Similarly, involvement of industry raises concerns about how to reconcile private and public interests in an adequate manner. For many examples in medicine (e.g., medications) it is clear that without industry involvement, diagnostic and therapeutic advances would not have been translated as quickly into clinical practice (Hawkins et al. 2009). However, the involvement of industry and commercialization brings challenges relating to trust (Chalmers and Nicol 2004), knowledge exclusion, trade secrets, and monopolies (Hong and Walsh 2009; Mitchell et al. 2011), intellectual property, conflict of interests, data sharing, informed consent, privacy, and confidentiality. Policy developments in the domain of human genetics should aim to maximize public benefit while allowing a level of intellectual property protection that is reasonably necessary to achieve that benefit. It should also be noted that while the inclusion of private interests and forms of incentive can be beneficial for fostering innovation and, thereby, widening access (albeit unequally), the balancing of such public and private interests can have a negative effect on levels of self-interest and altrustic motivations in society more generally and so would also be a reason for limiting any unqualified embrace of the private sector as a reliable means of promoting access for all in the longer term (Feeney 2012).

### Defining appropriate policies with regard to direct-to-consumer genetic testing

For over a decade, genetic testing companies have been marketing and selling genetic tests directly to consumers. This offer happens via the Internet, and often bypasses the traditional healthcare system and any healthcare professional involvement; due to these reasons, and more, DTC companies have been a source of controversy in academic and policy debates (Howard and Borry 2012). While the size of the DTC genetic testing market remains largely unknown (except for *23 and me*), it is probably relatively small. On the one hand, many companies that once sold DTC genetic tests have left the market. Various companies now collaborate with physicians and the traditional healthcare system, and have distanced themselves from a consumer-driven access model. On the other hand, as genetic testing has become much more affordable over the years and genetic testing has become more socially acceptable, various companies have remained in the field. A review of public and organizational policies on DTC indicated there was no uniform approach, with some professional organizations warning of harms and others supporting autonomous choice (Skirton et al. 2012). Although a new In Vitro Diagnostics (IVD) Regulation was voted at the European level and will come in to force in 2022, for regulators at the national level, the issue of DTC genetic testing will certainly remain on the agenda for the coming years. A first important policy question is the extent to which regulators want to intervene in the provision of genetic tests. Some have argued that “the embedding of genetic testing in a healthcare setting can ensure a context where due emphasis is being provided on the individualized medical supervision of patients, the presence of pre-test and post-test counseling, psychological evaluation and follow-up if appropriate and quality assurance of the tests performed” (Ayme et al. 2013). However, there are discussions regarding whether this should also apply to categories of tests that are labeled as “informational” or “recreational” or that do not offer any assessment of disease risk (Caulfield et al. 2015). Second, legislators can also impact the extent to which genetic tests are occurring within the scope of the healthcare system. Some countries have developed legislation that does not allow for direct access to genomic information, and imposes canalization of genetic tests through medical doctors or healthcare professionals (Kalokairinou et al. 2015). Third, various commentators have proposed a role for regulatory bodies in imposing and enforcing “truth in advertising” requirements in order to respond to the concerns relating to inaccurate information provision and subsequent consumer misunderstanding concerning the validity and utility of genomic information provided (EASAC and FEAM Working Group 2012). Fourth, the development of educational interventions targeted towards healthcare professionals and the general public in order to inform these groups about the lack of scientific validity and relevance of many of these DTC tests, has been suggested (EASAC and FEAM Working Group 2012). Finally, any regulation that would be developed to manage the DTC genetic testing market would always have to deal with the issue of (international) enforcement. It remains difficult to apply a regulatory control on an international market functioning through the Internet.

## Cross-cutting themes

### Maintaining trust

Various studies have shown that (public) trust is a cornerstone of participation in genomic research (Nobile et al. 2013). But trust is also fragile, and efforts need to be made at the level of information provision, consent procedures, and governance mechanisms in order for research participants to develop and maintain trust in research. Various studies have consistently found that publics have high levels of trust in universities and government research organizations. However, studies also show that trust in research diminishes if the research is funded by industry (Critchley and Nicol 2009). As knowledge of potential commercial access to genomic information is known to be a relevant consideration in the decision to participate in research, transparency regarding commercial use is ethically required (Caulfield et al. 2014). Informed consent is a mechanism that allows individuals to receive information to enable them to participle in research in a voluntary way. However, informed consent comes with its limitations and needs to be complemented by other governance mechanisms that might address societal concerns. In order to keep trust in technological innovations, it is also of crucial importance that appropriate safeguards are in place in order to protect individuals from inappropriate discrimination and stigmatization based on genetic information, and also human rights more broadly.

### Evidence building

Despite technological progress, there is still a wide gap between the DNA sequence data than can be generated and our ability to both interpret sequence variants and to derive possible health implications from sequence alterations in genes (Stemerding and Krom 2013). Although, clinical implementation of NGS technologies has proven to be valuable, various challenges remain before routine use of this technology can occur (Caleshu and Ashley 2016; Manolio et al. 2013). These include a lack of evidence and conflicting interpretations of benefit, a lack of institutional and clinical acceptance, and limited access to genomic medicine and testing. It also includes a lack of standards for genomic applications such as: integration of genomic results into electronic medical records and clinical decision support; follow-up of genotyped patients; outreach to at-risk family members; consent; understanding by patients, clinicians, and public; lack of access to comparison “control” sequence data and banking resources; and lack of research funding and reimbursement. Solutions to these problems are necessary in order to allow successful and responsible implementation into the clinical setting. Various commentators have also described the need for databases that include a comprehensive overview of genetic variants and related phenotypic information. This information should be accessible to various clinical groups worldwide who are involved in interpreting sequence data in clinical care and research. Many groups are currently doing this in isolation, and data sharing would benefit many patients around the world. Policies that reward or require data sharing should be developed (Cook-Deegan et al. 2013). Nevertheless, due attention should be paid to the legal requirements across jurisdictions that may concern cross-border sharing of genomic data. Furthermore, the views of the public need to be taken into account (Bentzen and Svantesson 2016; Majumder et al. 2016).

### Transferring knowledge to stakeholders

The full potential of the progress being made in genomics and related fields will not be realized unless the knowledge generated by such endeavors is translated into a usable format and transferred to all relevant stakeholders in society. The foremost focus should be on how best to inform all relevant stakeholders about the potential benefits and harms regarding accessing their genetic information from different sources, on developing and advertising best practice procedures, and on facilitating access to genetic knowledge in the most responsible and ethically acceptable way. As such, education must address all aspects of the technologies, including ethical issues and scientific validity. Rapid education and training in genomics is required for many different practitioners in the healthcare setting, from scientists and bioinformaticians carrying out diagnostic tests, to doctors in non-genetic specialties who may increasingly order such tests independently of clinical genetics services, to primary care clinicians such as GPs, specialist nurses, and midwives. Each stakeholder group will have different educational needs, and training must be pragmatic and reflect practical needs for certain information rather than an idealistic goal to upskill everyone significantly in all aspects of the field. Multi-national coordinated efforts (such as the Medgen Project or the Gen-Equip project) will be essential moving for forward in assisting with the mainstreaming and standardization of genomics into clinical care, as well as improving the visibility of genetics as a whole in the European context.

### Ensuring data security in clinical and research setting

Genetic data is being processed, stored and analyzed on an unprecedented scale thanks to decreasing costs; ~ 250,000 individual human genomes have been sequenced or are in progress thus far. Even with conservative estimates of doubling data quantities every 18 months, we will probably reach massive scale of data generation within the next decade. It is estimated that by 2025 between 1 and 25% of the eight billion humans worldwide will have had their genome sequenced (Stephens et al. 2015). The emerging possibilities for obtaining and storing genomic information and making it available to individuals, raise novel challenges with regard to the security of storage and processing. In many jurisdictions, genetic information is a type of information that receives special protection and information and communication technology (ICT) security measures need to meet those requirements. Platforms that host or analyze genetic information need to be equipped against security threats. In particular, the privacy of the data subjects, integrity of the databases and availability of the data to authorized users should be reinforced. Attention needs should be paid, not just to the development of a secure computing platform, but also to the security of potentially associated cloud providers, the legal protections cloud services enjoy in their respective jurisdictions, and to secure and controlled modes of access (Bentzen and Svantesson 2016). Unfortunately, genome data has a distributed data architecture where data acquisition is still not standardized. Instead it involves numerous heterogeneous formats (Costa 2012) which may raise questions about the data integrity and the adequate safeguards against unauthorized data uses (Knoppers et al. 2011). Moreover, the issues regarding the adequate storage and computational infrastructures in a widely accessible manner should be taken into consideration. (Eisenstein 2015).

## Conclusion

The expanded availability of genetic information is expected to influence the relationship between various parties, including healthcare professionals, individuals, families, research participants, researchers and industry. We have highlighted the main challenges arising from the availability of such information, and suggested areas for further research. In particular, we have underlined the significance of maintaining trust, building evidence, transferring knowledge to stakeholders, and ensuring data security in clinical and research settings, as the core elements to be respected in light of the expanded availability of genomic data and the identified challenges.

The identified challenges with regard to the expanded availability of genomic data require various stakeholders to engage in constructive discussions regarding the best practices for reporting test results, including reporting incidental findings and VUS. Given the familial implications of genetic data, it is essential to strike a balance between the rights, responsibilities, and autonomy of individuals dealing with their own genetic information, and the way these considerations intertwine with those of a family. Notably, in dealing with genetic data, it is essential to respect social values, such as fairness and justice.

Furthermore, developing adequate tools and guidelines in order to assist researchers in sharing genetic data is critical. Informed consent, privacy safeguards and oversight mechanisms should be improved in order to adequately address the concerns of individuals relating to data sharing and to ensure the ethical and legal footing of data sharing. Concurrently, educating both professionals and the general public could raise awareness regarding the significance of access to geno-mic data and assist in clarifying the roles and responsibilities of the parties involved.

The role of regulatory bodies in regulating various aspects of genetic testing within clinical and research settings is highlighted by this paper. In particular, regulating various aspects of commercial direct-to-consumer genetic testing, including advertisement of the products and the responsibilities of healthcare professionals in dealing with the results of such tests, are recognized as matters of concern.

The advancements in genomics and bioinformatic technologies urge an ongoing monitoring of the associated challenges, and the adequate addressing of them through robust policies. It is expected that this paper will direct future research and provide grounds for potential policy developments if needed.
